# Exosomes: composition, biogenesis, and mechanisms in cancer metastasis and drug resistance

**DOI:** 10.1186/s12943-019-0991-5

**Published:** 2019-04-02

**Authors:** Ladan Mashouri, Hassan Yousefi, Amir Reza Aref, Ali mohammad Ahadi, Fatemeh Molaei, Suresh K. Alahari

**Affiliations:** 1 Department of Genetics, Faculty of Science, Shahrekord University, Shahrekord, Iran; 2 Department of Biochemistry and Molecular Biology, LSUHSC School of Medicine, New Orleans, USA; 3 Department of Medical Oncology, Dana-Farber Cancer Institute, Harvard Medical School, Boston, MA 02115 USA; 4 Medical Genetics Research Center, Mashhad University of Medical Sciences, Mashhad, Iran

## Abstract

Tumor-derived exosomes (TDEs) participate in formation and progression of different cancer processes, including tumor microenvironment (TME) remodeling, angiogenesis, invasion, metastasis and drug-resistance. Exosomes initiate or suppress various signaling pathways in the recipient cells via transmitting heterogeneous cargoes. In this review we discuss exosome biogenesis, exosome mediated metastasis and chemoresistance. Furthermore, tumor derived exosomes role in tumor microenvironment remodeling, and angiogenesis is reviewed. Also, exosome induction of epithelial mesenchymal transition (EMT) is highlighted. More importantly, we discuss extensively how exosomes regulate drug resistance in several cancers. Thus, understanding exosome biogenesis, their contents and the molecular mechanisms and signaling pathways that are responsible for metastasis and drug-resistance mediated by TDEs may help to devise novel therapeutic approaches for cancer progression particularly to overcome therapy-resistance and preventing metastasis as major factors of cancer mortality.

**Electronic supplementary material:**

The online version of this article (10.1186/s12943-019-0991-5) contains supplementary material, which is available to authorized users.

## Introduction

Intercellular communication is crucial for cells to adapt to diverse intra- and extra-cellular alterations occurring in different processes such as embryonic development, response to injury, homeostasis, and other functions [1]. Different mechanisms for cell-cell communications from direct contact to distant interactions through body fluids and circulation are used for transmitting various signals. Transfer of biological mediators via microparticles and exosomes is a specific and extensively regulated transport mechanism [2, 3].

A plethora of evidence indicates that exosome-mediated factors can promote tumor initiation, metastasis, and therapy-resistance in cancer cells through cell-cell communication within the TME [3–5]. A normal niche is composed of various cells such as fibroblasts, endothelial and immune cells and a collection of extracellular matrix components, including cytokines, growth factors, and exosomes [6, 7]. Niche formation can lead to the survival and proliferation of the cancer stem cells (CSCs) and other tumor cells lead to a malignancy [8]. According to the cancer stem cell hypothesis, CSCs, a subpopulation of tumor cells, are responsible for the maintenance and recurrence of tumors [9]. Numerous studies confirmed that CSCs play key roles in the resistance of tumors to chemotherapy and radiotherapy [10]. The critical role of the TME in changing tumor behavior has been depicted by multiple studies revealing how the tumor microenvironment can change malignant behavior of the tumor cells [11]. It has been shown that exosomes influence different tumorigenic pathways in TME such as stemness, angiogenesis, metastasis, and hypoxia induced the EMT [12]. Moreover, other studies indicate that elimination of exosomes from the circulation inhibits tumor progression [13]. The initiation of tumorigenesis is not only based on the sufficient mutations to gain cancerous potential but also a functional alteration in the tumor microenvironment via different interactive mediators, such as exosomes [14–16]. Biological roles of TDEs as the microvesicles in the body fluids in TME progression is a subject of considerable interest in many studies [17]. Altogether, it is of paramount importance to elucidate exosome-mediated molecular mechanisms and signaling pathways that promote metastasis and therapy-resistance of the cancer cells in order to devise novel and more effective treatment strategies.

### Exosome discovery and composition

The experiment of Chargaff and West on the human plasma in 1946 determined that removal of pelleted plasma fraction after high-speed centrifugation inhibits plasma clotting [18]. Years later Peter Wolf discovered that these clotting suppressors are 20–50 nm vesicles derived from platelets [19]. In 1983, two papers published almost at the same time in *JCB* and *Cell* reported that transferrin receptors on reticulocytes interact with about 50 nm active vesicles that are derived from maturing sheep reticulocytes and secreted into the extracellular environment [5, 20]. Extracellular Vesicles (EVs) are classified into different groups of microvesicles, exosomes and apoptotic bodies based on morphological features and content [21]. Exosomes are intraluminal vesicles (ILV) of multivesicular bodies (MVB) which are 30–100 nm in diameter [5, 22]. High resolution analysis by electron microscopy together with advanced proteomic techniques disclosed the composition of exosomes secreted from different cells [5, 23].

Exosome contents not only mirror the composition of the donor cell but also reflect a regulated sorting mechanism [24]. A complex of various proteins including receptors, transcription factors, enzymes, extracellular matrix proteins, lipids, nucleic acids (DNA, mRNA, and miRNA) inside and on the surface of the exosomes constitute their content [25, 26]. Analysis of exosome protein composition revealed that some proteins specifically arise from cell and tissue of origin, and some are common among all exosomes [23]. Adhesion molecules such as CAMs, integrins, tetraspanins, MHC class I, II presented on B lymphocytes and dendritic cells together with transferrin receptors (TfR) on the surface of reticulocytes are among the typical examples of specific types of exosome proteins. On the other hand, a range of fusion and transferring proteins like Rab2, Rab7, flotillin and annexin, heat shock proteins such as Hsc70 and Hsc90, cytoskeleton proteins including actin, myosin, tubulin, and proteins such as Alix that mediate MVBs formation belong to non-specific protein types of exosomes [23, 27].

In addition, the lipid content of exosomes is cell-specific or conserved. Lipids not only have an important role in protecting exosome shape but also take part in exosome biogenesis and regulating homeostasis in the recipient cells [28–30]. The high density of lipids like lyosbisphosphatidic acid (LBPA) in the internal membrane of MVBs results in ILV formation and thus exosomes [29, 31]. LBPA interaction with Alix facilitates the sprouting of MVBs membrane inwardly [32]. Sphingomyelin, phosphatidylcholine and BMP are among factors that help distinguishing numerous types of vesicles. Different types of microvesicles have a similar content of sphingomyelin and phosphatidylcholine while sphingomyelin concentration is higher in exosomes. BMP is exclusive to endosomes as the origin of exosomes [33, 34]. Moreover, studies have shown that exosomes transferred to target cells could change the lipid composition of the recipient cells especially in cholesterol and sphingomyelin, and hence influence the cell homeostasis. ExoCarta is a database that involves all the published and unpublished data about exosome content, and it has more than 47,000 entries for protein, mRNA and lipid. Moreover, ExoCarta is a good resource for information that will be helpful for exosome characterization [35].

### Exosome biogenesis

**Fig. 1:**
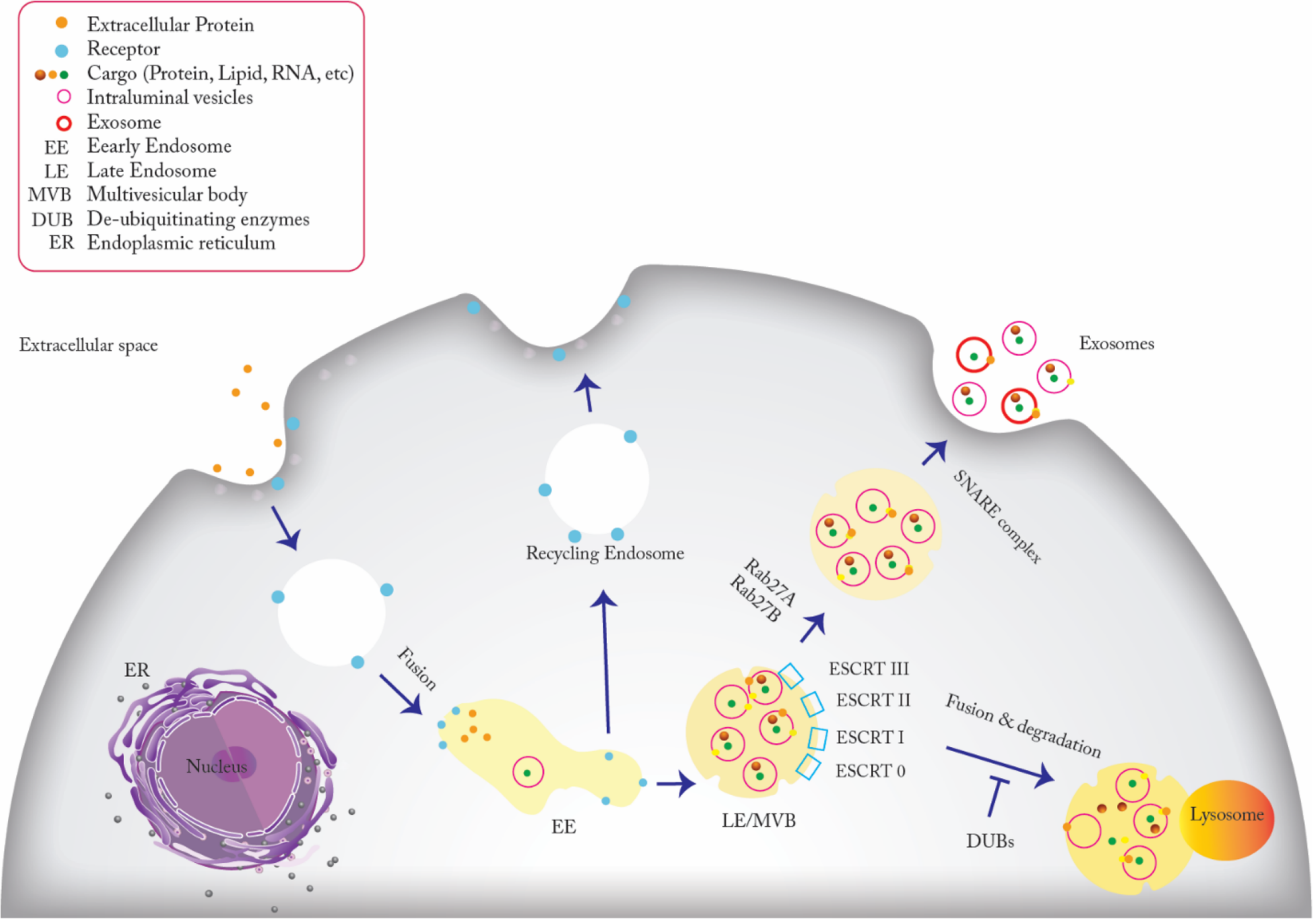
Exosome biogenesis and secretion within endosomal system. Early endosomes (EEs) are formed by the fusion of endocytic vesicles. Two pathways shown by the EEs: returning to the plasma membrane or conversion into LEs/ MVBs via inward budding of the membrane which pack the cargoes into ILVs. Protein sorting of ILVs can be ESCRT dependent or independent. ESCRT-0, ESCRT-I, ESCRT-II and ESCRT-III are four components of ESCRT machinery which ubiquitinate the substrates on the part of the inward budding endosomal membrane. Later, targeted ILVs are ready to be degraded within lysosome or rescue by DUBs. Rab27A and Rab27B are the essential mediators to lead the MVBs toward the cell periphery. Finally, SNARE complex helps fusion of MVBs with plasma membrane to release ILVs into the extracellular space which now are called exosomes

Activation of cell-specific receptors and the signaling pathways initiating exosome biogenesis are intensely regulated [36]. The fusion of primary endocytic vesicles is the first step of the early endosome (EE) formation [33]. Many incoming endocytic cargoes share their contents and membrane composition by combining the EEs in a clathrin- or caveolin-dependent or independent pathways [37]. EEs, undergo two pathways either by returning the cargo to the plasma membrane as the “recycling endosomes” or changing into “late endosomes” (LEs), also called MVBs (Fig. 1). Rab5 with its effector VPS34/p150 function as the key regulator of EV to LE conversion in the plasma membrane. Within several minutes after recycling their cargo to the cell membrane, ILV formation begins in EEs by inward membrane budding that leads to cargo sequestration and distribution into vesicles [33].

The protein sorting of ILVs is a highly regulated mechanism which is dependent on endosomal-sorting complex required for transport (ESCRT) machinery, and sometimes ESCRT-independent as well. ESCRT-0, ESCRT-I, ESCRT-II, and ESCRT-III are four complexes that constitute the components of ESCRT apparatus. At the beginning of the ESCRT-dependent pathway, there is a crossroad for cargo delivery, which is determined by the protein ubiquitin (ub) checkpoint. In the Ub-dependent pathway, all the ESCRT subunits are involved. ESCRT-0 is responsible for recognizing mono-ubiquitinated proteins by means of an HRS heterodimer and STAM1/2 [23, 38, 39]. HRS is a cytosolic protein associates with other proteins such as Eps15 and Clathrin. HRS-recruited-clathrin helps to encounter the ubiquitinated cargo [23, 40]. Next, ESCRT-I and ESCRT-II join the ESCRT-0 to make a strong recognition domain with high affinity to the ubiquitinated substrates on the part of the endosomal membrane where it will ultimately to sprout in [41]. Finally, ESCRT-III converges with the complex to pinch off the membrane and release the buds into the endosome [42]. ILVs are now targeted to be delivered to the lysosome for degradation unless the cargoes become de-ubiquitinated by de-ubiquitylating enzymes (DUBs) [43] (Fig. 1). Eventually, the components of the complex will be dissociated by the ATPase VPS4 with its co-factor VTA, and will be used for the next round [23].

As mentioned, Alix is a marker protein of exosomes with an important role in their biogenesis. Alix binds to ESCRT-III and deliver un-ubiquitinated cargoes to the ILVs by directly binding to the cargo like PAR1 or carrying the syndecans and tetraspanin CD63 indirectly [41]. It has been shown that direct interaction of syntenin (syndecan adaptor) with ALIX via three LYPX L motifs helps the budding of endosomal membrane [44]. The ESCRT-independent mechanism takes place in melanosomes, a lysosome/endosome related organelle, in melanocytes. Pmel17 is a melanosomal protein which engages its luminal domains along with lipids to contribute in ILV formation [45, 46]. Pmel17 is independent of ESCRT machinery and exists in clathrin-coated EEs. Tetraspanin CD63 is another protein which mediates the melanosome membrane invagination in an independent manner for that of ESCRT and ceramide [45, 47]. It has been demonstrated that protein PLP (proteolipid protein) is delivered to the ILVs from the lipid-enriched parts of an endosomal membrane such as cholesterol, ceramide, and sphingomyelin in an ESCRT independent manner. Since it has been proved that ceramide-rich parts of endosomes have a high susceptibility to inward budding, defection of sphingomyelinase (SMase or SMPD2), as a converter of sphingomyelin into ceramide leads to suppression of ILV formation [41].

After these primary steps, the MVBs arrive at their final intracellular destination. MVBs may be obliged to fuse with lysosomes and become degraded because of the ubiquitinated cargo they contain, or they may go toward the plasma membrane and release their ILVs to the extracellular environment [44, 48]. Some of the Rab family components such as Rab27A and Rab27B are the crucial mediators of exosome release, which is done by inducing MVBs transfer to the cell periphery and finally their fusion with the plasma membrane. [44, 49]. The soluble N-ethyl maleimide (NEM)-sensitive factor attachment protein receptor (SNARE) complex drives membrane fusion and thus exosome secretion. The fusion process begins with the interaction of synaptotagmin protein that acts as a calcium sensor and locates on MVBs and the plasma membrane protein, syntaxin. Then, accumulated MVBs dock the plasma membrane through trans-SNARE complex, which consists of V-SNARE and T-SNARE on MVB and plasma membrane respectively, leads to releasing of exosomes to the extracellular environment [50]. It is essential to understand exosome biogenesis (Fig. 1) and release because it will be helpful in developing new therapeutic strategies.

### TDEs play key role in tumor microenvironment remodeling

Fibroblasts, endothelial cells and infiltrating immune cells are the major cell types within a tumor microenvironment that interacts with tumor cells by exosome signaling. The consequences of these interactions depend on the origin of the exosomes determining the exosomal cargo [51, 52]. Stress conditions such as hypoxia, starvation, and acidosis increase exosome release from malignant cells leading to TME alteration and expansion which subsequently results in tumor progression [53, 54]. Thus, content analysis of the exosomes will reveal their function in TME progression in malignancies, and this will further lead to developing more efficient microvesicle-based strategies for cancer prognosis and therapy [55].

TDEs are capable of modulating tumor microenvironment and ECM by stimulation of the extracellular receptor signaling and disruption of cell adhesion formation [56–59]. Many types of integrins and integrin ligands have been reported to be carried by TDEs. Exosomal integrins participate in the initiation of cancer cell colonization and formation of a pre-metastatic niche [60]. In breast cancer cells, exosome-mediated transfer of miR-105 from metastatic breast cancer cells induces metastasis and vascular permeability in distant organs by downregulating and targeting tight junction protein ZO-1 and destroying the barrier function of endothelial monolayers [61]. Exosomal miR-25-3p from colorectal cancer cells regulates the expression of VEGFR2, ZO-1, Occludin and Claudin5 in endothelial cells via targeting KLF2 and KLF4. The miR-25-3p further promotes vascular leakiness and prepares pre-metastatic niche in distant sites, including liver and lung [62]. Additionally, cancer cell exosomes induce differentiation of many types of TME cells to cancer-associated fibroblasts (CAFs) that are dominant cell population of the tumor microenvironment in most cancers, thus exosomes playing a crucial role in ECM remodeling and TME reprogramming [63, 64]. Exosomes derived from CAFs contain different molecules such as growth factors and miRNAs that have different effects on the target cells of TME. Gemcitabine treatment of CAFs in pancreatic cancer induce the expression of Snail and miR-146a along with extended exosome secretion which stimulates epithelial cell proliferation [65]. It has been shown that CAF-derived exosomes supply nutrients for prostate and pancreatic cancer cells and drive them to glycolysis. On one hand, CAF-derived exosomes increase glucose uptake and inject metabolites to TCA cycle, on the other hand, they reduce the oxidative phosphorylation in mitochondria. These findings may help explain the rationale for ongoing cancer growth even under hypoxia or reduced nutrition sources [66].

### TDEs promote angiogenesis, invasion and metastasis

TDEs play important functions in different stages of invasion and metastasis cascade including angiogenesis, EMT, invasion, migration and establishment of a premetastatic niche [67, 68], which are described below.

### TDEs promote angiogenesis

**Fig. 2:**
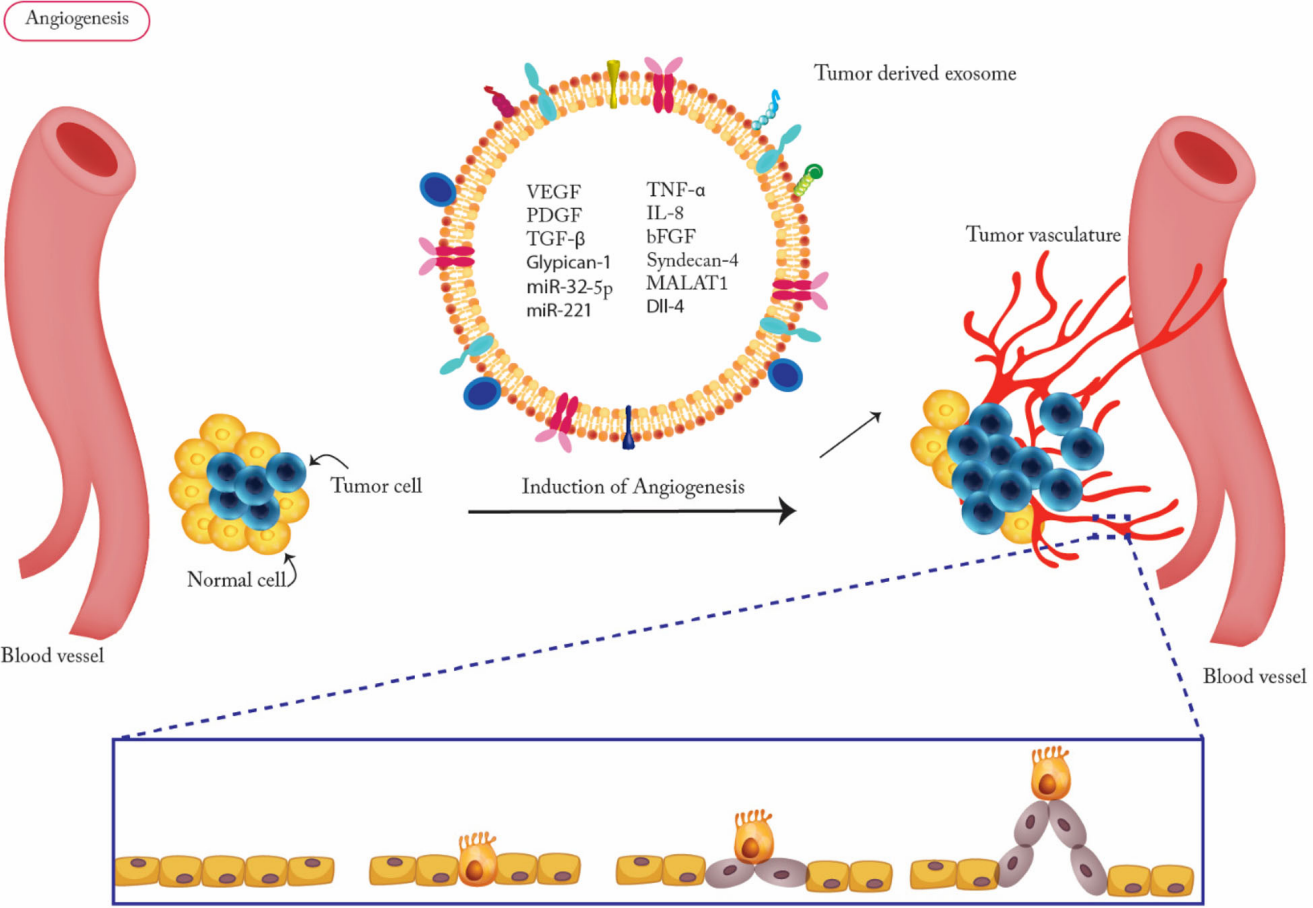
Tumor-derived exosomes promotes angiogenesis. Tumor derived exosomes promotes vascular formation. VEGF, Platelet-derived growth factor (PDGF), basic fibroblast growth factor (bFGF), transforming growth factor β (TGF-β), tumor necrosis factor α (TNF-α), syndecan-4, Glypican-1, interleukin-8 (IL-8), Malat1, Dil4 and some microRNAs are some of the angiogenic stimulatory factors which can be carried by tumor exosomes

Angiogenesis, a multi-step process by which tumors develop new vasculature, is essential for tumor growth and metastasis [69]. The vascular endothelial growth factor (VEGF)/VEGF receptor (VEGFR) signaling pathway is the most promising angiogenic target due to its key role in angiogenesis and tumor growth [70, 71]. Exosomes released by tumor cells is one of the main mechanisms that induce vascular formation. VEGF, fibroblast growth factor (FGF), Platelet-derived growth factor (PDGF), basic fibroblast growth factor (bFGF), transforming growth factor β (TGF-β), tumor necrosis factor α (TNF-α) and interleukin-8 (IL-8) are among main angiogenic stimulatory factors which are carried by TDEs. [72, 73] (Fig. 2). Exosomes from endothelial tip cells (the leading cells at the tips of vascular sprouts) contain high levels of delta-like-4 (Dll-4) protein that activates the Notch pathway within the neighboring microvascular endothelial cells resulting in the induction of capillary sprouts [74]. Cell line- or plasma-derived exosomes from a variety of human tumors including, glioblastoma, pancreatic and nasopharyngeal carcinomas, are known to be potent inducers of angiogenesis in vitro and in vivo [75, 76]. In vitro analysis of glioblastoma as an acutely angiogenic tumor type revealed that glioblastoma-derived exosomes contain high levels of miR-221, proteoglycans glypican-1 and syndecan-4 that increase revascularization via enhancing proliferation and formation of endothelial cells and tubules [77]. TDEs containing MALAT1 (metastasis-associated lung adenocarcinoma transcript 1) which is associated with angiogenesis and metastasis in epithelial ovarian cancer (EOC), induce pro-angiogenesis gene expression in human umbilical vein endothelial cells (HUVECs) [78] (Fig. 2). Also TDEs from head and neck squamous cell carcinoma (HNSCC) cell lines induced angiogenesis through reprogramming and modulation of endothelial cells [79]. Human breast-cancer-derived exosomes promote vascular leakiness in the lung by upregulating a subset of S100 proteins and activating Src kinase signaling [80]. Growing information on exosome roles in angiogenesis indicates that microvesicles are functional tools for suppressing endothelial cell migration, their phenotype alteration, and vessel sprouting in the solid tumors.

### Exosome-mediated induction of EMT

**Fig. 3:**
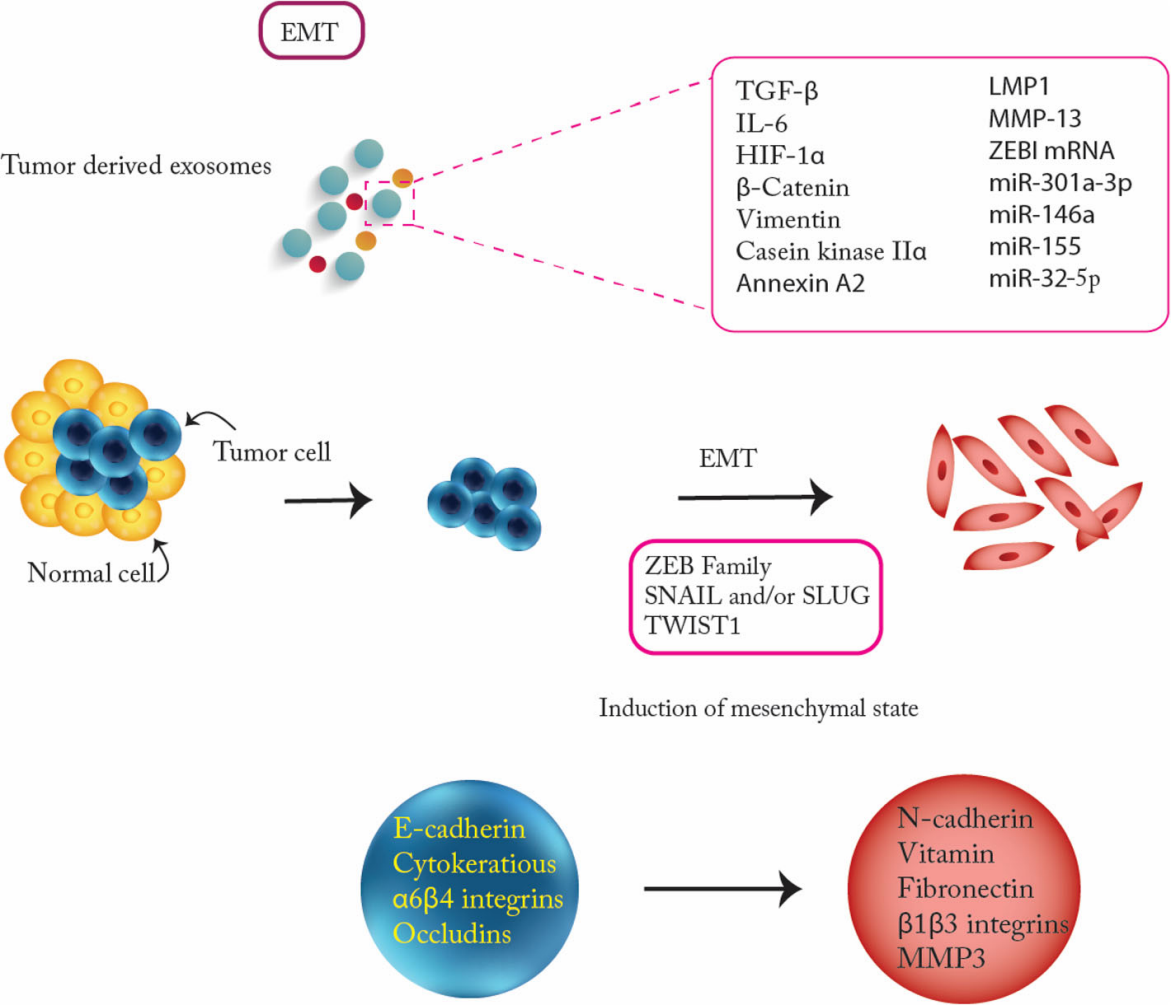
Tumor derived exosomes promote Epithelial Mesenchymal Transition (EMT) and Invasion. EMT initiators include TGF-β, HIF1α, β-catenin, IL-6, β-catenin or Vimentin, Casein Kinase and several miRNAs are among the essential EMT promoting factors that are carried by the tumor derived exosomes. Zeb family members are the main players in EMT transition

The more aggressive malignant tumor cells become, the more likely they are to migrate to distant sites. To help facilitate this, a new metastatic niche is needed in the distant site, and tumor epithelial cells must go through epithelial to mesenchymal transition (EMT) process [81]. During EMT, ECM is degraded and tumor epithelial cells change morphologically and functionally to become more invasive. TGF-β, HIF1α, β-catenin, IL-6, caveolin-1 or vimentin and nucleic acids like EMT-inducer miRNAs are among the essential EMT elements that are carried by the TDEs (Fig. 3). Tumor cells lose their epithelial features and gain mesenchymal properties by losing E-Cadherin and cell polarity while gaining N-cadherin, twist, snail, and vimentin. As a result of EMT epithelial cells become aggressive and attain stem cell-like properties [82–84]. Increased levels of proteins such as casein kinase II α and annexin A2 in the lumen of metastatic bladder cancer cells derived from exosomes are associated with EMT [85]. Exosomal LMP1, an EBV-encoded primary oncogene, contributes to severe metastatic features of NPC by upregulating EMT which is accompanied by the expression of cancer stem cell markers [86]. Hypoxia-mediated TDEs enriched with miR-301a-3p in pancreatic cancer cells enhance transition of macrophages into the M2 phenotype. This transition promotes migration and EMT of pancreatic cancer cells which is due to activation of PTEN/PI3K pathway [87]. Matrix metalloproteinase (MMP) 13-containing exosomes facilitate the metastasis of nasopharyngeal cancer (NPC) cells (an endemic type of head and neck cancer) and it is through induction of EMT [88]. These reports suggest that exosomes play a pivotal role in regulating EMT.

### Role of TDEs in promoting migration, invasion and metastasis

It has been shown that Rab27b-mediated exocytic release of HSP90 exosomes from metastatic breast cancer cells activate MMP2. This activation leads to degradation of ECM components, release of growth factors, and promotion of cancer cell invasion [89]. Through initiating PI3K/AKT and MAPK signaling pathways and upregulation of MMPs, TDEs from metastatic HCC cell lines promote the migration and invasion of non-motile hepatocyte cells. This enhancement is partly due to shuttling various tumor progression factors including RNAs and proteins such as caveolins, MET proto-oncogene, and S100 family members [90]. Also in a study by Peinado et al, it was reported that exosome-derived MET oncoprotein released by highly metastatic melanoma cells increased the metastatic behavior of bone marrow (BM) progenitors and induce vascular leakiness and inflammation at pre-metastatic sites through activating MET pathway [91]. This lead to the up-regulation of many factors including heat-shock proteins, S100a8, S100a9 and TNF-α. Urothelial cells treated with bladder cancer exosomes showed an increased expression in several mesenchymal markers, including α-SMA, S100A4 and snail, and associated with decreased expression of epithelial markers such as E-cadherin and β-catenin, thus promoting EMT [92] (Fig. 3). An experiment by Sanchez et al revealed that differentially expressed miRNAs via Prostate Cancer (PCa) bulk and CSCs, and exosomes collaboratively cause PCa progression and metastasis. Transfection of normal prostate fibroblasts with exosomal miR-21-5p, miR-100-5p and miR-139-5p increased the expression of MMP-2, MMP-9 and MMP-13 and RANKL lead to promoting their migration [93]. Exosomal miR-494 and miR-542-3p taken up by lymph node stromal cells and lung fibroblasts contribute to target cell reprogramming by downregulation of cadherin- 17 (Cdh17) and upregulation of MMP2, MMP3, and MMP14 establishing a premetastatic niche [9]. Exosomal miR-222-3p stimulates invasion, and anti-anoikis behavior in NSCLC cells by targeting SOCS3 (Suppressor of cytokine signaling 3) which is a negative regulator of the JAK/STAT signaling pathway. Furthermore, increased level of exosomal miR-222-3p in serum of the NSCLC patients was associated with poor prognosis [94, 95]. TDEs containing miR-222 enhanced the migration and invasion ability of breast cancer cells through down-regulation of PDLIM2- (a tumor suppressor) which lead to activation of NF-κB signaling pathway [96]. Studies on the metastatic niche have endorsed that high amounts of integrins and MMPs increase matrix and basement membrane degradation [97]. Evidence suggests that TDEs transfer to new metastatic niche (pre-metastatic niche) based on targeting specific recipient cells. Membrane proteins and lipids are engaged in organotropic metastasis of tumor exosomes. For example, primary tumor-derived exosomes carrying α6β4 and α6β1 integrins destined to lung while αvβ5 home to the liver. Furthermore, in the new destination, exosomes undertake roles for establishment and progression of the pre-metastatic niche [67]. Normal cells within the pre-metastatic organ influenced by exosomal miRNAs that are responsible for transformation and angiogenesis and metastatis [98]. In HCC, exosomes transferring SMAD Family Member 3 (SMAD3) protein and mRNA that potentiated adhesive ability in the recipient HCC cells by enhancing SMAD3 signaling in these cells [99]. In summary, TDEs play a significant role in regulating metastatic niche through various proteins and microRNAs. (Fig. 3).

### TDEs promote therapy-resistance

In normal cells, exosomes function in removing unfavorable biomolecules, however in cancer cells this could be hijacked in the context of cancer chemotherapy. It has been shown that exosomes play a key role in all steps of EMT multistage process, from the induction of invasive phenotype to distant metastasis [67, 84]. Drug-resistant cancer cells are able to pack the chemotherapeutic agents in exosome and shuttle anti-cancer drugs out of the tumor cell [100]. Furthermore, delivery of exosomal cargo containing miRNA, mRNA and proteins to cancerous cells is associated with tumor drug resistance [101]. Since exosomes are used as a genetic exchange vector in the tumor microenvironment, drug-resistant tumor cells hijack this mechanism to confer resistance to sensitive cells [102]. Therapy-resistance mechanisms mediated by exosomes are summarized in Additional file 1: Table S1.

### Therapy-resistance through induction of EMT

The role of EMT in tumor drug resistance has been widely investigated. Multiple EMT-mediated signaling pathways mostly anti-apoptotic pathways and the up-regulation of drug efflux pumps, characteristics similar to CSCs, are involved in drug resistance [103]. In MCF-7 and MDA-MB-231 breast cancer cells, exosome-mediated miR-155 induces chemoresistance through increasing EMT markers and targeting TGF-β, FOXO-3a and C/EBP-β mRNA [104]. In ovarian cancer cells, TDEs promote platinum resistance through alteration of TGF- β/SMAD signaling and up-regulation of EMT markers [105]. In gastric cancer cells, miR-155-5p causes paclitaxel resistance through inducing EMT, and targeting GATA binding protein 3 (GATA3) and tumor protein p53-inducible nuclear protein 1 (TP53INP1) [106]. Moreover, oncogenically transformed human bronchial epithelial cell (HBEC) have been shown to induce gemcitabine and cisplatin resistance in NSCLC cells by promoting EMT [107]. Exosomes from epithelial ovarian cancer A2780 platinum-resistant cells achieve resistance through promoting EMT [105]. Exosomes derived from glioblastoma cells contain PTPRZ1-MET fusion proteins which confer temozolomide resistance through EMT [108].

### TDEs induce therapy-resistance through promoting anti-apoptotic pathways

Apoptotic pathway inhibitors have been shown to sensitize tumor cells to chemotherapy. Both acquired or intrinsic resistance to chemotherapies, frequently make tumor cells resistant from undergoing sufficient levels of apoptosis which results in cancer survival and dismal treatment outcome [109]. LncRNA-SNHG14 in Her2+ breast cancer induces exosome-mediated trastuzumab resistance by targeting the apoptosis regulator Bcl-2 / BAX signaling pathway [110]. Elevated p-AKT levels as a result of exosome- mediated miR-21 delivery to NSCLC cells increase gefitinib resistance [111]. In HCC cells, exosomes containing miR-32-5p cause multi-drug resistance through activating PI3K/AKT pathway and inhibiting PTEN by promoting angiogenesis and EMT [112]. Moreover, exosomes from cisplatin-resistant oral squamous cell carcinoma (OSCC) cells target PTEN and PDCD4 which results in therapy-resistance [113]. In colon cancer, cetuximab-resistant RKO colon cancer cells through downregulating PTEN and increasing phosphorylation of AKT levels induce cetuximab resistance [114]. In addition, PLX-4720 BRAF inhibitor–resistant melanoma cells have shown resistance to BRAF inhibitor by activating of PI3K/AKT signaling and escaping from MAPK pathway [115]. Recently it has been revealed that hepatocellular carcinoma (HCC) derived exosomes containing miR-221 lead to sorafenib resistance by modulating caspase-3 and apoptosis inhibition [116].

### Therapy-resistance via drug efflux or drug sequestration

Several multidrug resistance (MDR) mechanisms lead to chemotherapy-resistance including attenuation in uptake of drugs soluble in water and increase in energy-dependent efflux of hydrophobic drugs. P-glycoprotein (P-gp) is among the main anticancer pumping transporters [117]. Recent studies on the treatment of human ovarian carcinoma with cisplatin (CDDP) has shown that exosomal malformed protein sorting and releasing lead to more CDDP export and drug resistance behavior. Increase in MRP2, ATP7A, and ATP7B CDDP transporters expression enhances CDDP exclusion accumulated in lysosomes [100]. TDEs from gemcitabine -resistant pancreatic cancer cells induce chemoresistance through trapping P-gp and MRP-5 or to allow gemcitabine to flow back to the microenvironment [118]. Tumor-associated macrophages (TAM) derived exosomes contain miR-365 which upregulates the triphospho-nucleotide and induces the enzyme cytidine deaminase leads to inactivation of gemcitabine pool in pancreatic ductal adenocarcinoma (PDAC) cancer cells [119]. Exosomes derived from human breast cancer cells (MCF7/WT) contain UCH-L1 and P-gp proteins that are capable of inducing adriamycin resistance by up-regulating the expression of P-glycoprotein [120]. Adriamycin-resistant breast cancer cells contain GSTP1 which detoxifies several anti-cancer drugs by conjugating them with glutathione [121]. Increased expression of GSTP1 decreased the apoptotic rate in recipient cells. It has been suggested that GSTP1 regulates cell survival by inactivating NF-κB and pERK pathways in cervical cancer [122]. HER2-overexpressing exosomes contain an intact HER2 molecule which counteract trastuzumab-based therapy by binding to the antibody [123]. Exosomes directly or indirectly regulate drug efflux pumps which in turn affect drug resistance.

### Therapy-resistance through signal transduction alteration

The EGFR, PI3K/AKT, PTEN and mTORC signaling pathways play critical roles in tumor progression and drug resistance. The aberration of these pathways has been associated with resistance to chemotherapies [124]. TDEs and exosomes from surrounding cells including CAFs, TAMs and tumor-associated vasculature are capable of altering various signal transduction pathways and their regulations [125, 126]. Exosomes from A549 cells, a cisplatin (CDDP) resistant human lung adenocarcinoma cell line induce therapy-resistance via up-regulation of mTOR expression [127]. In addition, heparanase containing exosomes from myeloma cells enhance activation of ERK signaling pathway [128]. In another study, colorectal cancer cells secrete exosomes which are able to cause 5-FU and Oxaliplatin resistance via activating the Wnt/β-catenin pathway by promoting the stabilization and nuclear translocation of β-catenin [129]. Triple-negative breast cancer (TNBC) cells released exosomes induce docetaxel and gemcitabine resistance in non-tumorigenic breast cells (MCF10A). Gene and miRNA expression profiling revealed that, resistance mediated by up-regulation of mostly the PI3K/AKT, MAPK, and HIF1A pathways in MCF10A cells [130]. These reports suggest that exosomes alter various signal transduction pathways to regulate drug resistance.

### Therapy-resistance through immune cell modulation

Recent studies have shown that there is one main mechanism through which tumor-derived exosomes modulate the tumor microenvironment by repressing the immune effector cells response and inducing immune suppressor cells [67, 131]. Another exosome-mediated mechanism that helps cancer cells evade the immune effector cells is by using decoy [132]. In addition, neutralizing antibody-based drugs via TDEs is another mechanism to decrease the drug’s effects [132]. In chronic lymphocytic leukemia, exosomal CD20 has been confirmed to intercept the anti-CD20 antibody rituximab and reduce its efficacy due to attenuation of the antibody deposition on target cells [133]. During treatment of HER2-overexpressing breast carcinoma cell lines with trastuzumab (anti-Her2 antibody), these cells release exosomes containing high levels of the Her-2 molecule which is less active than originated cells. Trastuzumab in tumor microenvironment can interact with Her-2 expressing exosomes in order to reduce its activity on the original tumor cells [123]. In addition, HER2+ breast cancer cells confer trastuzumab resistance by secreting exosomes containing immunosuppressive cytokine TGFb1 and the lymphocyte activation inhibitor PDL1- [134]. Recently, it has been shown that exosomal content includes proteins, RNA and DNA that contribute to the tumor immunity modulation [135]. TDEs were proved to be able to promote therapy-resistance via carrying unshielded RN7SL1 which activate the pattern recognition receptor (PRR) retinoic acid-inducible gene I (RIG-I) [136].

### Cancer stem cell-derived exosomes promote drug-resistance

Currently, many studies on therapeutic strategies have focused on CSCs due to their function in tumor initiation, therapy-resistance, and disease recurrence [137]. Studies on breast cancer stem cells (BCSCs) have shown that after cancer treatment, Notch, Wnt, and Hedgehog signaling pathways promote CSCs survival and proliferation. These pathways control self-renewal and differentiation of the BCSCs. Under environmental stress such as radiation and hypoxia, the levels of various CSCs survival and growth factors including TNF-α, TGF-β, PDGF, CXCL12, MMP, and HIF elevate within the TME [138]. CSC-derived exosomes maintain the stemness features within the TME via transferring their cargo. This cargo consists of Hedgehog, Wnt, β-catenin and other CSC specific mRNAs and proteins for CSCs to sustain their self-renewal and other stemness characteristics, therefore, enhancing resistance to different cancer therapies [139]. Integrin is a transmembrane receptor with α and β subunits which controls conformational changes and participate in cell migration, differentiation, proliferation, and survival [140]. Many types of integrins and integrin ligands have been reported to be carried by TDEs. Exosomal integrins participate in the initiation of cancer cell colonization and formation of a pre-metastatic niche [60]. Integrins are essential in the maintenance of stem cell phenotype and behavior. Moreover, it has been shown that integrin β1 is a crucial resistance factor in head and neck cancer radiotherapy and Erlotinib treatment in lung cancer [141].

### Exosomes in cancer therapy

Exosomes could be a functional means of drug-delivery in cancer therapy specifically because they are non-toxic and non-immunogenic. Exosome-based delivery approach of adriamycin and paclitaxel has been used for targeted cancer therapy and resulted in minimal immunogenicity and toxicity [142, 143]. In addition, many different cell types can generate exosomes. Moreover, exosomes can permeate through tumor cells at a higher rate than liposomes. Another advantage of exosomes is that they can target specific cells and tissues through specific proteins, therefore, they could deliver drugs targeted to cancer cells. Furthermore, exosomes are small in size, therefore, they can easily pass through various barriers such as the blood-brain barrier [144]. In addition, exosomes can be manipulated easily to increase their efficiency in targeting cancer cells [145]. Increasing evidence has shown that tumor derived exosomal RNA from the blood and other bodily fluids can be used as biomarkers in cancer screening and diagnosis [146].

**Fig. 4:**
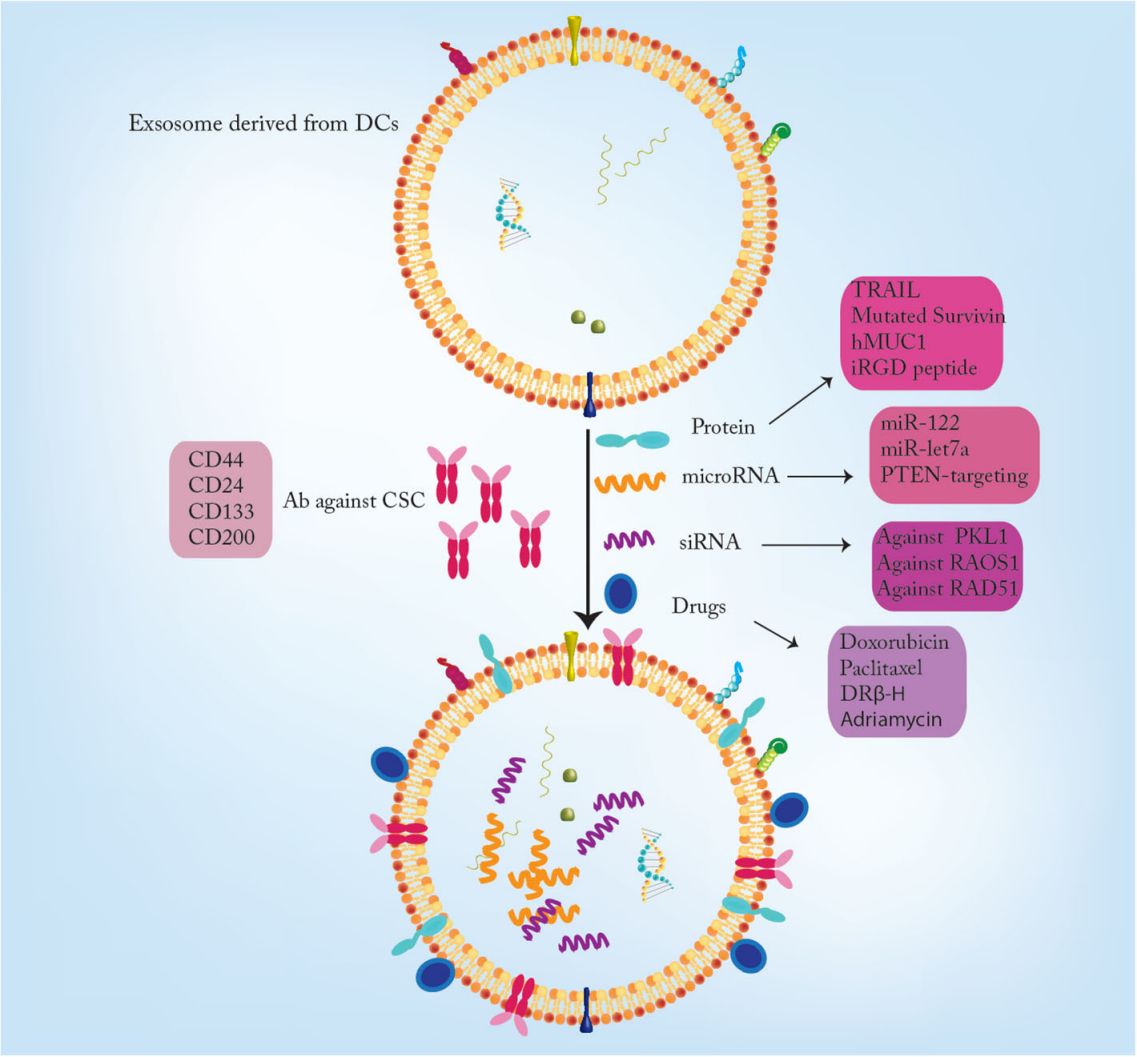
Exosome application in cancer therapy. Exosomes derived from DCs could be used to deliver Anti-cancer drugs such as chemotherapeutic agents and functional RNAs including siRNA and microRNA. Moreover, peptides also could be delivered to the target cancer cells especially CSCs. Such examples are TRAIL and mutated survivin. The specific markers of CSCs such as CD44, CD24, CD133, and CD200 can be used as targets for exosomes contain the antibodies against these proteins

Bioengineered exosomes have been used to deliver anti-cancer drugs and functional RNAs to cancer cells including CSCs in a cell-specific manner. Targeting CSCs by exosomes is one of the promising approaches in developing cancer therapies. To create modified exosomes, the donor cells are engineered to produce specific proteins on the exosome’s membrane. Specific makers of CSCs such as CD44, CD24, CD133, and CD200 can be used as targets for exosomes [147] (Fig. 4).

Dendritic cells (DCs) play key roles in both primary and secondary immune response, thus, exosomes derived from DCs are potential candidates for specific cancer therapy. Astrocyte-derived exosomes transfer PTEN-targeting miRNAs to metastatic tumor cells which suppress PTEN expression. Following loss of PTEN in invading tumor cells, CCL2 secretion elevates IBA1-expressing myeloid cell recruitment and metastatic seeding [148]. In addition, the human embryonic kidney cells have been modified to secrete exosomes which express GE11 peptide. These exosomes are capable of binding to the Epidermal Growth Factor Receptor (EGFR), which is expressed by many tumor cells with epithelial origin. In addition, these exosomes can be used to deliver specific non-coding RNAs such as miRNA let7a to the target cells [149] (Fig. 4).

Recently, the utilization of chemotherapeutic drug-loaded exosomes have been shown to be a novel approach in cancer therapy. The exosomes have improved the anti-tumor effects of these drugs. Paclitaxel-loaded exosomes are used for the treatment of several cancers including prostate, lung and pancreatic cancer [150, 151]. Moreover, Doxorubicin-loaded exosomes in a mouse model of colon cancer resulted in the reduction of tumor size [152]. Furthermore, in the mouse model of inflammatory tumors, the exosomes containing doxorubicin had an efficient anti-tumor effects. Doxorubicin-loaded exosomes also showed a great efficiency in targeting breast cancer cells [142]. The exosomes that are derived from DCs could be used to present tumor antigens to the Naïve T cells to induce a response to the tumor cells. The exosomes that are derived from two MHC type-distinct mouse cell lines were used to express tumor antigen human mucin1 (hMUC1). These exosomes have been shown to induce the immune system effectively [153] (Fig. 4).

Exosomes could serve as potential vehicles to transport functional proteins to cancer cells in a specific and targeted fashion. Tumor Necrosis Factor (TNF)-related Apoptosis-inducing Ligand (TRAIL) is a protein which induces apoptosis in cancer cells. The exosomes that are modified to secrete TRAIL showed great efficacy in inducing apoptosis in tumor cells and resulted in the tumor size reduction [154]. Survivin is an anti-apoptotic protein that is highly expressed in many types of cancers. An inactivating mutation (T34A) in Survivin impairs its normal function. Exosomes transferring this type of mutated Survivin promotes apoptosis in pancreatic adenocarcinoma cells [155] (Fig. 4).

Furthermore, exosomes have been used to deliver therapeutic RNAs such as siRNA and miRNAs to cancer cells. miRNA-122 induces chemosensitivity in hepatocellular carcinoma. Exosomes derived from adipose mesenchymal stem cells are used to deliver miR-122 promoted chemosensitivity of the HCC cells [156]. The siRNA against RAD51 was delivered to Hela and, HT1080 cells by exosomes resulted in massive cell death in recipient cancer cells [157]. The exosomes that are derived from MSC and HEK293 were used to deliver siRNA that target PKL-1 in bladder cancer cells and showed the reduced expression of polo-like kinase 1 (PLK-1) [158] (Fig. 4).

### Concluding remarks

In this review, we elaborated upon exosome biogenesis and the main mechanisms for exosome-mediated metastasis and chemoresistance. Understanding the molecular mechanisms underlying exosome biogenesis, metastasis and chemoresistance will aid in designing novel therapeutics targeting exosome-mediated tumorigenesis metastasis, and chemoresistance. Exosomes are versatile and critical intercellular connectors which follow a regulated mechanism for transferring biomolecules. Since exosomes are multi-purpose vehicles with organotropic behavior, aberration of their normal function provides favorable mechanism for tumors to utilize. Tumor-derived exosomes facilitate tumor growth, angiogenesis, invasion, formation of the pre-metastatic niche, and increase the therapy-resistance behavior of the tumor cells. Since exosomes are involved in many pathophysiological conditions, there has been a drive in research to utilize exosomes as a treatment strategy by loading them with therapeutic agents including functional proteins, miRNAs and various chemotherapeutics. There is still a long way to overcome the barriers presently impeded exosome-based therapeutic strategies for cancer therapy. Nevertheless, several key aspects regarding the underlying mechanisms of exosome-mediated crosstalk in tumor microenvironment, distant cell interactions, exosome heterogeneity, molecular mechanisms responsible for resistance and metastasis, have become increasingly apparent. Finally, designing different research approaches in this new vast area of study based on the tumor context will direct our understandings of exosomal-mediated therapy-resistance in different cancers and the translation of these findings to the clinical realm will provide a novel and effective treatment modality for future cancer patients.

## Additional file

Additional file 1: **Table S1.** Exosome mediated Therapy resistance mechanisms. (DOCX 58 kb)

## Abbreviations

5-FU: Fluorouracil
BAX: Bcl-2 Associated X
Bcl-2: B-cell lymphoma 2
BCSCs: Breast cancer stem cells
BRAF: Serine/threonine-protein kinase B-raf
C/EBP-β: CCAAT-enhancer-binding protein β
CAF: Cancer associated fibroblast
CAFs: Cancer-associated fibroblasts
CAMs: Cell adhesion molecules
CCL2: C-C Motif Chemokine Ligand 2
CD: Cluster of differentiation
CDDP: Cisplatin
CSCs: Cancer stem cells
CXCL12: C-X-C Motif Chemokine Ligand 12
DC: Dendritic cell
DUBs: De-ubiquitylating enzymes
EEs: Early endosomes
EGFR: Epidermal Growth Factor Receptor
EMT: Epithelial-mesenchymal transition
ERK: Extracellular signal–regulated kinase
ESCRT: Endosomal-sorting complex required for transport
EVs: Extracellular Vesicles
FOXO-3a: Forkhead box O3
GATA3: GATA Binding Protein 3
GSTP1: Glutathione S-transferase Pi 1
HBEC: Human bronchial epithelial cell
HCC: Hepatocellular carcinoma
HEK: Human embryonic kidney
Hela: Henrietta Lacks
HER2: Human epidermal growth factor receptor 2
hMUC1: human mucin1
IBA1: Ionized calcium-binding adapter molecule 1
ILV: Intraluminal vesicles
LBPA: Lyosbisphosphatidic acid
LEs: Late endosomes
LncRNA: Long non-coding RNA
MAPK: Mitogen-activated protein kinases
MCF10A: Michigan Cancer Foundation-10A
MCF7-WT: Michigan Cancer Foundation − 7 wild-type
MDR: Multidrug resistance
MET: Met Proto-Oncogene
MHC: Major histocompatibility complex
miR: miRNA
miRNA: MicroRNA
MMP: Matrix metalloproteinases
MRP2: Multidrug resistance-associated protein 2
MRP5: Multidrug resistance-associated protein 5
MSC: Mesenchymal stem cell
mTOR: Mammalian target of rapamycin
mTORC: mammalian target of rapamycin complex
MVB: Multivesicular bodies
NEM: N-ethyl maleimide
NF-κB: Nuclear Factor kappaB
NSCLC: Non-small-cell lung carcinoma
OSCC: Oral squamous cell carcinoma
PDAC: Pancreatic ductal adenocarcinoma
PDCD4: Programmed Cell Death 4
PDGF: Platelet-derived growth factor
P-gp: P-glycoprotein
PI3K: Phosphoinositide 3-kinase
PLK-1: Polo-like kinase 1
PLP: Proteolipid protein
PRR: Pattern recognition receptor
PTEN: Phosphatase and tensin homolog
PTPRZ1: Protein Tyrosine Phosphatase, Receptor Type Z1
RIG-I: Retinoic acid-inducible gene I
RN7SL1: RNA, 7SL, Cytoplasmic 1
siRNA: Small interfering RNA
SMase or SMPD2: Sphingomyelinase
SNHG14: Small Nucleolar RNA Host Gene 14
TAM: Tumor-associated macrophage
TDEs: Tumor-derived exosomes
TfR: Transferrin receptors
TGF-β: Transforming growth factor beta
TME: Tumor microenvironment
TNBC: Triple Negative Breast Cancer
TNF: Tumor Necrosis Factor
TNF-α: Tumor necrosis factor alpha
TP53INP1: Tumor Protein P53 Inducible Nuclear Protein 1
TRAIL: TNF-related Apoptosis-inducing Ligand
Ub: Ubiquitin
UCH-L1: Ubiquitin carboxy-terminal hydrolase L1
Wnt: Wingless-related integration site
