# Supplementary material for: Exosomes: composition, biogenesis, and mechanisms in cancer metastasis and drug resistance
Source: Mol Cancer. 2019 Apr 2;18:75. doi: 10.1186/s12943-019-0991-5 (PMC6444571; doi:10.1186/s12943-019-0991-5)
Supplement: Supplementary file 1 — Table S1. Exosome mediated Therapy resistance mechanisms. (DOCX 58 kb) [file 12943_2019_991_MOESM1_ESM.docx]

| Exosome content | Cell origin of exosome | Tumor type | Function | Mechanism | Reference |
| --- | --- | --- | --- | --- | --- |
| 5’-triphosphate exosome RNA | Stromal cell | Breast cancer | Cisplatin and radiation resistance | Activation of STAT1 signaling through RIG-I receptor cooperating with juxtacrine NOTCH3 signaling | [1] |
| HER2 | HER-2 overexpressing breast cancer cells | Breast cancer | Trastuzumab resistance | Shielding target cells from antibody dependent cell cytotoxicity (ADCC) mediated by NK cells by  sequestration of trastuzumab | [2] |
| - | MSCs (Mesenchymal stem cells) | Gastric cancer | 5-fluorouracil (5-FU) resistance | Enhancing the anti-apoptotic ability by activating CaM-Ks/Raf/MEK/ Erk pathway, and promoting MDR, MRP and LRP expression | [3] |
| Somatic mutated SMAD4 protein and mRNA | Platinum-resistant epithelial ovarian cancer (EOC) cells | Ovarian cancer | Carboplatin resistance | Mediating EMT by Up-regulation of miR-21 (pri-miR-21) | [4] |
| MDR (multiple drug resistance)-1/P-gp | Breast cancer cells | Breast cancer | Docetaxel resistance | Promoting drug efflux | [5] |
| Survivin | Breast cancer cells | Breast cancer | Paclitaxel resistance | lengthening the survival of paclitaxel-treated cells | [6] |
| Transient receptor potential channel (TRPC5) | Chemoresistant breast cancer cells | Breast cancer | Adriamycin resistance | Stimulating P-gp production in the recipient cells through a Ca2+- and NFATc3-mediated mechanism | [7] |
| miRNAs | Chemoresistant prostate cancer cells | Prostate | Taxanes resistance | Regulating of the translation of AR, PTEN and TCF4 genes | [8] |
| miR-146a and Snail mRNA | Cancer associated fibroblast (CAFs) | Pancreatic adenocarcinoma (PDAC) | Gemcitabine resistance | Enhancing cell proliferation and EMT by increasing the levels of Snail mRNA and miR-146a | [9] |
| microRNA-96 | Malignant lung cancer cells | Lung cancer | Cisplatin resistance | Promoting cell proliferation and migration by targeting LMO7 | [10] |
| GSTP1 | Adriamycin-resistant cells | Breast cancer | Anthracycline/taxane-based neoadjuvant chemotherapy | Detoxification of drugs by conjugating them with glutathione to decrease apoptotic rate | [11] |
| PTPRZ1-MET fusion (ZM fusion) | Glioblastoma cell | Glioblastoma | Temozolomide resistance | Altering gene expression and inducing EMT | [12] |
| miR-221 | Hepatocellular carcinoma cells (HCC) | HCC | Sorafenib resistance | Apoptosis inhibition by modulating caspase-3 expression level | [13] |
| miR-1246 | Paclitaxel-resistant ovarian cancer cells | Ovarian cancer | Paclitaxel | Inhibition of 3’UTR Caveolin-1 which directly inhibits PDGFRβ kinase activity and increase in p-gp expression | [14] |
| miR-451, miR-326 | Chemoresistant breast cancer cells | Breast cancer | Doxorubicin | Increasing expression of P-gp | [15] |
| miR-221/222 | Tamoxifen-resistant breast cancer cells | ER positive breast cancer | Tamoxifen | Increasing cell proliferation by down regulating P27 and ERα protein levels | [16] |
| lncRNA PART1 | Gefitinib-resistant ESCC cells | Esophageal squamous cell carcinoma (ESCC) | Gefitinib | Binding of PART1 to miR-129 to facilitate Bcl-2 expression | [17] |
| miR-21 | CAFs | Ovarian cancer | Paclitaxel | Apoptosis inhibition by targeting APAF1 and inducing invasion and metastasis by regulation of MMP1 expression indirectly via targeting genes in the TGF signaling pathways | [18] |
| Androgen-receptor splice variant 7 messenger RNA (AR-V7) | Resistant cells | Prostate cancer | Enzalutamide and abiraterone | Inhibition of the androgen receptor may lead to reciprocal up-regulation of other oncogenic pathways, such as the PI3K– AKT pathway | [19] |
| linc-RNA-ROR | HCC cells | hepatocellular cancers (HCCs) | Sorafenib or doxorubicin | Cell apoptosis inhibition and expression enhancement of tumor-initiating cells by selective enrichment of linc-RNA-ROR via TGFb related pathway | [20] |
| STAT3 and  FAS | Hypoxic ovarian cancer cells | Ovarian cancer | Cisplatin | Increasing cisplatin efflux, cell migration/invasion | [21] |
| miR-145 and miR-34a | 5-FU resistant human colon cancer DLD-1 cells | Colon cancer | 5-FU resistance | - | [22] |
| - | Gemcitabine resistant pancreatic cancer cells | Pancreatic cancer | Gemcitabine resistance | P-gp and MRP-5 trap gemcitabine and allow gemcitabine to flow back to the microenvironment | [23] |
| downregulated miR-100–5p | Cisplatin- resistant human lung adenocarcinoma (LAC) cells | Lung adenocarcinoma | Cisplatin resistance | mTOR expression was reverse regulated by  miR-100–5p | [24] |
| miR-155 | Docetaxel and paclitaxel resistant breast cancer cells | Breast cancer | Doxorubicin and paclitaxel resistance | Increase in EMT markers and targeting TGF-β, FOXO-3a and C/EBP-β mRNA | [25] |
| Ephrin type-A receptor 2 (EphA2) | Gemcitabine resistant pancreatic cancer cells | Pancreatic cancer | Gemcitabine resistance | EXO_EphA2 expression | [26] |
| LncRNA PART1 | Esophageal squamous cell carcinoma cells | Esophageal squamous cell carcinoma | Gefitinib resistance | Sponging miR-129 and activating the Bcl-2 signaling pathway | [27] |
| lncRNA-SNHG14 | HER2+ breast cancer cells | HER2+ breast cancer | Trastuzumab resistance | Targeting the apoptosis regulator Bcl-2/BAX signaling pathway | [28] |
| Transcripts of miR155 | Pancreatic cancer cells | Pancreatic cancer | Gemcitabine resistance | Increasing the levels of superoxide dismutase 2 (SOD2) and catalase (CAT; ROS-detoxifying enzymes) and downregulation of DCK (gemcitabine-metabolizing gene) | [29] |
| miR‑21 | NSCLC cells | Gefitinib resistance  NSCLC | Gefitinib resistance | Elevated p‑Akt levels and promotion activation of Akt | [30] |
| Elevated oncogenic miR-889, oncogenic mRNAs, and proteins of the proteasome pathway, Notch, Jak-STAT, and cell cycle pathways | Cell line models of nervous system cancer | Nervous system cancer | Radiation resistance | Promotion of cell cycle, growth/survival | [31] |
| H19 | NSCLC cell lines | NSCLC | Gefitinib resistance | - | [32] |
| miR-214 | NSCLC cell lines | NSCLC | Gefitinib resistance | - | [33] |
| Elevated S100A16 | SCLC cells | Enhanced resistance to apoptosis under stressful conditions  SCLC | resistance to apoptosis under stressful conditions | Inducing the elevation and translocation from the cytoplasm to the nucleus of S100A16 in the recipient SCLC cells and modulating the mitochondrial function | [34] |
| MGMT mRNA | Glioma cells | Glioma | Temozolomide (TMZ) resistance | Translation of exogenous exosomal MGMT mRNA | [35] |
| miR-32-5p | HCC cell lines | Hepato-Cellular Carcinoma | 5-FU, Oxaliplatin, gemcitabine and sorafenib resistance | Activating PI3K/Akt pathway by suppressing PTEN and inducing multidrug resistance and promoting angiogenesis and epithelial-mesenchymal transition (EMT) | [36] |
| Wnts glycoproteins | Colorectal cancer (CRC) cells | Colorectal cancer | 5-FU and Oxaliplatin resistance | Activate the Wnt/β-catenin pathway by promoting the stabilization and nuclear translocation of β-catenin | [37] |
| miR‑155‑5p | Paclitaxel‑resistant gastric cancer cells | Gastric cancer | Paclitaxel resistance | By inducing EMT and  targeting GATA binding protein 3 (GATA3) and tumor protein p53‑inducible nuclear protein 1 (TP53INP1) | [38] |
| lncRNA urothelial carcinoma‑associated 1 (UCA1) | Colorectal cancer cetuximab‑resistant cells | Colorectal cancer | Cetuximab resistance | - | [39] |
| miR-365 | Tumor-associated macrophages (TAM) | Pancreatic ductal adenocarcinoma (PDAC) | Gemcitabine resistance | miR-365 impair activation of gemcitabine by upregulation of the triphospho-nucleotide pool in cancer cells and the induction of the enzyme cytidine deaminase inactivates gemcitabine | [40] |
| Heparanase | Myeloma cells | Myeloma | - | Enhancing  their heparan sulfate degrading activity and leading to activation of ERK signaling and an increase in shedding of the  -syndecan-1 proteoglycan | [41] |
| DNMT1  Transcripts | Ovarian cancer cells | Ovarian cancer | Cisplatin resistance | - | [42] |
| miR-1246 | Metastatic breast cancer cells | Breast cancer | Docetaxel and gemcitabine resistance | Suppressing the  expression level of its target gene, Cyclin-G2 (CCNG2) | [43] |
| miR-221 | Glioma cells | Glioma | Temozolomide resistance | Targeting DNM3 | [44] |
| SOD2 and CAT (ROS detoxifying  Transcripts) | Pancreatic cancer cells | Pancreatic cancer | Gemcitabine resistance | Upregulation of SOD2 and CAT (ROS detoxifying  genes), and downregulation of DCK (gemcitabine-metabolizing gene) | [29] |
| miR-21 | Cisplatin-resistant OSCC cells | Oral squamous  cell carcinoma (OSCC) | Cisplatin resistance | Targeting  PTEN and PDCD4 in oral squamous cell  Carcinoma | [45] |
| ZEB1 mRNA | Oncogenically transformed lung cells mesenchymal HBECs | NSCLC cells | Gemcitabine and cisplatin resistance | Promoting EMT | [46] |
| - | Cetuximab-resistant colon cancer cells | Colon cancer | Cetuximab resistance | Downregulation of PTEN and increasing phosphorylated Akt levels | [47] |
| PDGFRβ | (PLX-4720) BRAF inhibitor–resistant melanoma cells | Melanoma | BRAF inhibitor–resistance | Activation of PI3K/AKT signaling and escaping from  MAPK pathway BRAF inhibition | [48] |
| miR-155 overexpression | Gemcitabine-resistant PDAC cells | Pancreatic Adeno Carcinoma Cells | Gemcitabine resistance | Inducing anti-apoptotic pathway | [49] |
| Immunosuppressive cytokine TGFb1 and the lymphocyte activation inhibitor PD-L1 | HER2-positive breast cancer cells | Breast cancer | Trastuzumab resistance | Decreasing the antibody-dependent cell cytotoxicity (ADCC) mediated by trastuzumab | [50] |
| UCH-L1 and P-gp proteins | Human breast cancer cells | Breast cancer | Adriamycin resistance | Up-regulated expression of P-gp by activating the MAPK/ERK signaling pathway | [51] |
| - | A2780  platinum-resistant EOC lines | Epithelial Ovarian Cancer | Platinum resistance | Increased epithelial to mesenchymal transition (EMT) | [52] |

**Table 1.** Therapy-resistance mechanisms mediated by TDEs are summarized here.

1. Boelens, M.C., et al., *Exosome transfer from stromal to breast cancer cells regulates therapy resistance pathways.* Cell, 2014. **159**(3): p. 499-513.

2. Marleau, A.M., et al., *Exosome removal as a therapeutic adjuvant in cancer.* 2012. **10**(1): p. 134.

3. Ji, R., et al., *Exosomes derived from human mesenchymal stem cells confer drug resistance in gastric cancer.* Cell Cycle, 2015. **14**(15): p. 2473-83.

4. Crow, J., et al., *Exosomes as mediators of platinum resistance in ovarian cancer.* J Oncotarget, 2017. **8**(7): p. 11917.

5. Lv, M.-m., et al., *Exosomes mediate drug resistance transfer in MCF-7 breast cancer cells and a probable mechanism is delivery of P-glycoprotein.* J Tumor Biology, 2014. **35**(11): p. 10773-10779.

6. Kreger, B.T., et al., *The Enrichment of Survivin in Exosomes from Breast Cancer Cells Treated with Paclitaxel Promotes Cell Survival and Chemoresistance.* Cancers (Basel), 2016. **8**(12): p. 111.

7. Ma, X., et al., *Essential role for TrpC5-containing extracellular vesicles in breast cancer with chemotherapeutic resistance.* Proc Natl Acad Sci U S A, 2014. **111**(17): p. 6389-94.

8. Li, J., et al., *Exosome-derived microRNAs contribute to prostate cancer chemoresistance.* Int J Oncol, 2016. **49**(2): p. 838-46.

9. Richards, K.E., et al., *Cancer-associated fibroblast exosomes regulate survival and proliferation of pancreatic cancer cells.* Oncogene, 2017. **36**(13): p. 1770-1778.

10. Wu, H., et al., *Circulating exosomal microRNA-96 promotes cell proliferation, migration and drug resistance by targeting LMO7.* J Cell Mol Med, 2017. **21**(6): p. 1228-1236.

11. Yang, S.J., et al., *Predictive role of GSTP1-containing exosomes in chemotherapy-resistant breast cancer.* Gene, 2017. **623**: p. 5-14.

12. Zeng, A., et al., *Tumour exosomes from cells harbouring PTPRZ1–MET fusion contribute to a malignant phenotype and temozolomide chemoresistance in glioblastoma.* %J Oncogene, 2017. **36**(38): p. 5369.

13. Fornari, F., et al., *In hepatocellular carcinoma miR-221 modulates Sorafenib resistance through inhibition of caspase-3 mediated apoptosis.* J Clinical Cancer Research, 2017: p. clincanres. 1464.2016.

14. Kanlikilicer, P., et al., *Exosomal miRNA confers chemo resistance via targeting Cav1/p-gp/M2-type macrophage axis in ovarian cancer.* EBioMedicine, 2018. **38**: p. 100-112.

15. Jaiswal, R., et al., *Microparticle-associated nucleic acids mediate trait dominance in cancer.* FASEB J, 2012. **26**(1): p. 420-9.

16. Wei, Y., et al., *Exosomal miR-221/222 enhances tamoxifen resistance in recipient ER-positive breast cancer cells.* Breast cancer research, 2014. **147**(2): p. 423-431.

17. Kang, M., et al., *Exosome-mediated transfer of lncRNA PART1 induces gefitinib resistance in esophageal squamous cell carcinoma via functioning as a competing endogenous RNA.* Journal of Experimental

Clinical Cancer Research

2018. **37**(1): p. 171.

18. Yeung, C.L.A., et al., *Exosomal transfer of stroma-derived miR21 confers paclitaxel resistance in ovarian cancer cells through targeting APAF1.* Nature communications, 2016. **7**: p. 11150.

19. Antonarakis, E.S., et al., *AR-V7 and resistance to enzalutamide and abiraterone in prostate cancer.* New England Journal of Medicine, 2014. **371**(11): p. 1028-1038.

20. Takahashi, K., et al., *Extracellular vesicle‐mediated transfer of long non‐coding RNA ROR modulates chemosensitivity in human hepatocellular cancer.* FEBS open Bio, 2014. **4**(1): p. 458-467.

21. Dorayappan, K.D.P., et al., *Hypoxia-induced exosomes contribute to a more aggressive and chemoresistant ovarian cancer phenotype: a novel mechanism linking STAT3/Rab proteins.* Oncogene, 2018: p. 1.

22. Akao, Y., et al., *Extracellular disposal of tumor-suppressor miRs-145 and-34a via microvesicles and 5-FU resistance of human colon cancer cells.* International journal of molecular sciences, 2014. **15**(1): p. 1392-1401.

23. Muralidharan-Chari, V., et al., *Microvesicle removal of anticancer drugs contributes to drug resistance in human pancreatic cancer cells.* Oncotarget, 2016. **7**(31): p. 50365.

24. Qin, X., et al., *Cisplatin-resistant lung cancer cell–derived exosomes increase cisplatin resistance of recipient cells in exosomal miR-100–5p-dependent manner.* International journal of nanomedicine, 2017. **12**: p. 3721.

25. Santos, J.C., et al., *Exosome-mediated breast cancer chemoresistance via miR-155 transfer.* Scientific reports, 2018. **8**(1): p. 829.

26. Fan, J., et al., *Chemoresistance Transmission via Exosome-Mediated EphA2 Transfer in Pancreatic Cancer.* THERANOSTICS, 2018. **8**(21): p. 5986-5994.

27. Kang, M., et al., *Exosome-mediated transfer of lncRNA PART1 induces gefitinib resistance in esophageal squamous cell carcinoma via functioning as a competing endogenous RNA.* Journal of Experimental & Clinical Cancer Research, 2018. **37**(1): p. 171.

28. Dong, H., et al., *Exosome-mediated transfer of lncRNA‑SNHG14 promotes trastuzumab chemoresistance in breast cancer.* International journal of oncology, 2018. **53**(3): p. 1013-1026.

29. Patel, G.K., et al., *Exosomes confer chemoresistance to pancreatic cancer cells by promoting ROS detoxification and miR-155-mediated suppression of key gemcitabine-metabolising enzyme, DCK.* British journal of cancer, 2017. **116**(5): p. 609.

30. Jing, C., et al., *Exosome‑mediated gefitinib resistance in lung cancer HCC827 cells via delivery of miR‑21.* Oncology letters, 2018. **15**(6): p. 9811-9817.

31. Mrowczynski, O.D., et al., *Exosomes impact survival to radiation exposure in cell line models of nervous system cancer.* Oncotarget, 2018. **9**(90): p. 36083.

32. Lei, Y., et al., *Tumor‑released lncRNA H19 promotes gefitinib resistance via packaging into exosomes in non‑small cell lung cancer.* Oncology Reports, 2018. **40**(6): p. 3438-3446.

33. Zhang, Y., M. Li, and C. Hu, *Exosomal transfer of miR-214 mediates gefitinib resistance in non-small cell lung cancer.* Biochemical and biophysical research communications, 2018. **507**(1-4): p. 457-464.

34. Xu, Z.-H., et al., *Brain microvascular endothelial cell exosome–mediated S100A16 up-regulation confers small-cell lung cancer cell survival in brain.* The FASEB Journal, 2018: p. fj. 201800428R.

35. Yu, T., et al., *Delivery of MGMT mRNA to glioma cells by reactive astrocyte-derived exosomes confers a temozolomide resistance phenotype.* Cancer letters, 2018. **433**: p. 210-220.

36. Fu, X., et al., *Exosomal microRNA-32-5p induces multidrug resistance in hepatocellular carcinoma via the PI3K/Akt pathway.* Journal of Experimental & Clinical Cancer Research, 2018. **37**(1): p. 52.

37. Hu, Y.-B., et al., *Exosomal Wnt-induced dedifferentiation of colorectal cancer cells contributes to chemotherapy resistance.* Oncogene, 2018: p. 1.

38. Wang, M., et al., *Paclitaxel‑resistant gastric cancer MGC‑803 cells promote epithelial‑to‑mesenchymal transition and chemoresistance in paclitaxel‑sensitive cells via exosomal delivery of miR‑155‑5p.* International journal of oncology, 2019. **54**(1): p. 326-338.

39. Yang, Y.-n., et al., *Predictive role of UCA1-containing exosomes in cetuximab-resistant colorectal cancer.* Cancer cell international, 2018. **18**(1): p. 164.

40. Binenbaum, Y., et al., *Transfer of miRNA in Macrophage-Derived Exosomes Induces Drug Resistance in Pancreatic Adenocarcinoma.* Cancer research, 2018. **78**(18): p. 5287-5299.

41. Bandari, S.K., et al., *Chemotherapy induces secretion of exosomes loaded with heparanase that degrades extracellular matrix and impacts tumor and host cell behavior.* Matrix Biology, 2018. **65**: p. 104-118.

42. Cao, Y.L., et al., *Exosomal DNMT1 mediates cisplatin resistance in ovarian cancer.* Cell biochemistry and function, 2017. **35**(6): p. 296-303.

43. Li, X.J., et al., *Exosomal MicroRNA MiR-1246 promotes cell proliferation, invasion and drug resistance by targeting CCNG2 in breast cancer.* Cellular Physiology and Biochemistry, 2017. **44**(5): p. 1741-1748.

44. Yang, J.-K., et al., *Exosomal miR-221 targets DNM3 to induce tumor progression and temozolomide resistance in glioma.* Journal of neuro-oncology, 2017. **131**(2): p. 255-265.

45. Liu, T., et al., *Exosomes containing miR-21 transfer the characteristic of cisplatin resistance by targeting PTEN and PDCD4 in oral squamous cell carcinoma.* Acta biochimica et biophysica Sinica, 2017. **49**(9): p. 808-816.

46. Lobb, R.J., et al., *Exosomes derived from mesenchymal non‐small cell lung cancer cells promote chemoresistance.* International journal of cancer, 2017. **141**(3): p. 614-620.

47. Zhang, S., et al., *Exosomes promote cetuximab resistance via the PTEN/Akt pathway in colon cancer cells.* Brazilian Journal of Medical and Biological Research, 2018. **51**(1).

48. Vella, L.J., et al., *Intercellular Resistance to BRAF Inhibition Can Be Mediated by Extracellular Vesicle–Associated PDGFRβ.* Neoplasia, 2017. **19**(11): p. 932-940.

49. Mikamori, M., et al., *MicroRNA-155 controls exosome synthesis and promotes gemcitabine resistance in pancreatic ductal adenocarcinoma.* Scientific reports, 2017. **7**: p. 42339.

50. Martinez, V.G., et al., *Resistance to HER2-targeted anti-cancer drugs is associated with immune evasion in cancer cells and their derived extracellular vesicles.* Oncoimmunology, 2017. **6**(12): p. e1362530.

51. Ning, K., et al., *UCH‐L1‐containing exosomes mediate chemotherapeutic resistance transfer in breast cancer.* Journal of surgical oncology, 2017. **115**(8): p. 932-940.

52. Crow, J., et al., *Exosomes as mediators of platinum resistance in ovarian cancer.* Oncotarget, 2017. **8**(7): p. 11917.
